# Detailed Understanding of the Electrochemistry and Oxygen Reduction Activity of La_1–*x*_Ca_*x*_MnO_3_ Obtained by In Situ XAS and XES

**DOI:** 10.1021/acselectrochem.5c00541

**Published:** 2026-04-14

**Authors:** Veronica Celorrio, Haoliang Huang, Shusaku Hayama, David J. Fermin, Andrea E. Russell

**Affiliations:** † Diamond Light Source Ltd., Diamond House, Harwell Campus, Didcot OX11 0DE, UK; ‡ School of Chemistry and Chemical Engineering, University of Southampton, Highfield, Southampton SO17 1BJ, UK; § 641635 Songshan Lake Materials Laboratory, Dongguan 523808, China; ∥ School of Chemistry, University of Bristol, Cantocks Close, Bristol BS8 1TS, UK

**Keywords:** Mn-based perovskite oxides, oxygen reduction reaction, X-ray absorption spectroscopy, X-ray emission spectroscopy, redox−structure relationships

## Abstract

Understanding the interplay between redox behavior and structural stability is crucial for the development of transition metal oxides in electrocatalysis. In this work, we use both X-ray absorption spectroscopy (XAS) and X-ray emission spectroscopy (XES) to investigate the electrochemical response of Mn-based perovskite oxides (La_1–*x*_Ca_*x*_MnO_3_) under oxygen reduction reaction (ORR) conditions. This dual approach enables tracking of changes in both the oxidation state and local coordination environment. Mn Kβ XES data show that oxidation-state changes are reversible, despite a shift in transition potentials across a range of compositions, including CaMnO_3._ In contrast, Mn K-edge EXAFS analyses reveal that while LaMnO_3_ retains structural integrity, CaMnO_3_ undergoes irreversible structural changes at low potentials, associated with the collapse of the perovskite framework. Intermediate compositions show partially reversible structural behavior. This decoupling of redox reversibility and structural instability, a picture only accessible through the use of XAS and XES, provides critical insight into the complex behavior of these materials under operational conditions. Additionally, our analysis shows that Mn­(II) formation is only detected in CaMnO_3_ at potentials more negative than 0.4 V (vs RHE). The ORR onset is associated with Mn­(IV) reduction, while peroxide formation correlates with an increased Mn­(III)/Mn­(IV) ratio, supporting a 2e^–^ + 2e^–^ reduction pathway. This study demonstrates the power of XAS and XES analyses to disentangle electronic and structural dynamics, providing a more complete understanding of activity–stability relationships in perovskite electrocatalysts.

## Graphical abstract

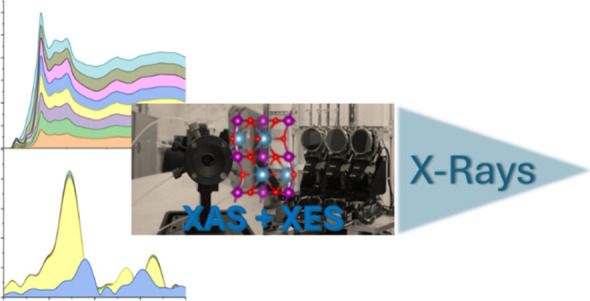

## Introduction

Understanding the redox behavior and local atomic structure of transition metal-based electrocatalysts under operational conditions is critical to the rational design of materials for energy conversion processes.^1–7^ Spectroscopic techniques capable of resolving subtle changes in both the oxidation state and coordination environment are therefore essential.^8–11^ Among these, X-ray absorption spectroscopy (XAS) and X-ray emission spectroscopy (XES) have emerged as highly complementary tools for providing detailed, element-specific insight into the electronic and structural dynamics of catalytically active centers.^12–16^

XAS offers quantitative information on both the oxidation state and local coordination environment of the absorbing atom.^17^ The X-ray absorption near-edge structure (XANES) region is sensitive to changes in oxidation state, local symmetry, and unoccupied density of states. On the other hand, the extended X-ray absorption fine structure (EXAFS) region provides access to bond distances, coordination numbers, and disorder parameters, enabling reconstruction of the local atomic geometry around the absorber. Interpretation of XANES alone can sometimes be complicated by overlapping spectral features or the influence of structural distortions on edge position.^18,19^

To overcome these limitations and refine the interpretation of oxidation-state changes, XES serves as a valuable complement to XAS. In particular, nonresonant Kβ XES is sensitive to the spin state and oxidation state of the metal center, offering a more direct probe of the valence electronic configuration.^20^ Because Kβ XES originates from core-level transitions (3p → 1s) influenced by the 2p–3d exchange interaction and hence 3d density, it provides oxidation-state information that is less perturbed by geometric effects compared to XANES.^21^ By providing complimentary information, XAS and XES allow for the cross-validation of redox assignments and build a more comprehensive understanding of the electronic structure under operando or in situ conditions.

In electrocatalysis, studies using both XAS and XES offer a particularly powerful approach for investigating the electronic and structural dynamics of active materials under operational conditions.^9,22^ Transition metal centers often undergo redox transitions during electrochemical cycling, which are closely tied to catalytic activity, stability, and degradation mechanisms. By integration of oxidation-state information obtained from Kβ XES with local structural parameters derived from EXAFS, it becomes possible to distinguish between reversible/irreversible changes associated with catalysis and structural degradation. This combined methodology also enables the identification and tracking of catalytically relevant intermediates, providing a comprehensive understanding of the material’s behavior during reaction.

In this work, we employ XAS and XES in separate experiments to investigate the electrochemical behavior of Mn-based perovskite oxides under oxygen reduction conditions. By analyzing the data from both Mn K-edge EXAFS and Kβ XES measurements, we disentangle the electronic and structural contributions to redox activity, providing detailed insight into the reversibility of Mn oxidation states and coordination changes. This multimodal spectroscopic strategy highlights the potential of XAS and XES to serve as diagnostic platforms for probing the functional dynamics of complex oxide electrocatalysts.

## Experimental Section

### Sample Preparation

La_1–__*x*_Ca_*x*_MnO_3_ perovskite materials were synthesized employing an ionic liquid-based route, as described in previous works.^23,24^ A dispersion of Mn­(NO_3_)_2_ (0.5 mL, 0.1 M in H_2_O) and 0.5 mL of the corresponding A-site metal nitrate (0.5 mL, 0.1 M in H_2_O) in 1-ethyl-3-methylimidazolium acetate (1 mL) was prepared and kept under stirring conditions. After the mixture was heated at 80 °C for 4 h to ensure that all water was removed, cellulose (100 mg) was added, and the mixture was stirred for 20 min. The gel-like mixture was calcined for 2 h at 700 °C. For CaMnO_3_, a dwell temperature of 850 °C was required to achieve a crystalline structure. As investigated previously, this preparation method yields nanoparticles in the range of 30 nm of phase-pure rhombohedral (*R-3cH* space group) LaMnO_3_, while the introduction of Ca^2+^ promotes the orthorhombic *Pnma* space group.^24^

### Rotating Ring-Disk Electrochemical Measurements

A three-electrode cell was used to conduct the electrochemistry experiments by using a rotating ring-disk electrode (RRDE) fitted to an ALS rotation controller and connected to a CompactStat bipotentiostat (Ivium). The RRDE electrode consisted of a 4 mm glassy carbon disk surrounded by a Pt ring. The collection efficiency was experimentally determined to be 0.4. A graphite rod and a Hg/HgO (in 0.1 M NaOH) were used as counter and reference electrodes, respectively. Measurements were recorded in 0.1 M KOH solution saturated with either purified Ar or O_2_. The sample loading (per unit area of the glassy carbon disk) on the electrode surface was 250 μg_OXIDE_ cm^–2^, 50 μg_VULCAN_ cm^–2^, and 50 μg_NAFION_ cm^–2^.

### Preparation of Electrodes and In Situ XAS and XES Measurements

Button electrodes were prepared by painting the catalyst ink onto a carbon paper support with a loading of 0.5 mg of Mn cm^–2^. All measurements were collected in situ, using our custom-designed electrochemical cell connected to an IVIUM potentiostat.^25^ Measurements were collected in N_2_-purged 0.1 M KOH electrolyte. XES and high-energy-resolution fluorescence-detected (HERFD-XANES) measurements were conducted at the beamline I20-scanning, of the Diamond Light Source,^26,27^ operating with a ring energy of 3 GeV and at a current of 300 mA. The beamline is equipped with an in-house-designed four-bounce scanning Si(111) monochromator, and harmonic rejection was achieved by using two Rh-coated mirrors. An X-ray emission spectrometer was based on a 1 m diameter Rowland circle operating in the Johann configuration in the vertical plane. Three Ge(440) analyzers were used to collect the Mn Kβ emission signal for the experiment.^27^ To minimize sample illumination, a fast beam shutter was automatically inserted when no data were acquired during motor motions (monochromator or sample positioning), and we checked that no radiation damage occurred by recording spectra as a function of time. In the XANES analysis, the edge position was determined as the energy corresponding to half of the normalized edge step. The XES spectra have been normalized with respect to their total area using the range of 6465–6497 eV.

Separate extended X-ray absorption fine structure (EXAFS) measurements were carried out at room temperature at the B18 beamline of Diamond Light Source.^28^ The monochromator comprises Si(111) crystals operating in Quick EXAFS mode. Calibration of the monochromator was carried out using a Mn foil, and XAFS spectra were recorded in fluorescence mode at the Mn K-edge (6539 eV) using a Canberra 36-elements monolithic planar Ge pixel array detector. The spectra were aligned by using the Mn foil response. The data was analyzed using the Athena and Artemis softwares.^29^

Conventional fluorescence XANES and HERFD-XANES were collected by using different electrochemical acquisition protocols. Because HERFD-XANES+XES required longer counting times, the electrode was held at each applied potential for significantly longer durations, which may result in a greater reduction in the Mn species if the process is kinetically slow. Also, the HERFD-XANES+XES series was recorded over a larger number of potential steps than that of the conventional XANES series. As a result, the total illumination time and potential history differ between the two datasets; therefore, their apparent potential-dependent spectral changes cannot be compared quantitatively on the basis of the X-ray absorption edge position. Nevertheless, both datasets are included in this manuscript, as the information obtained is complementary, and taken together, a more detailed picture of the distribution of the oxidation states (XES data) and structure (EXAFS data) is obtained.

## Results and Discussion

### Electrocatalytic Behavior of (La, Ca)­MnO_3_ Oxides

Figure 1a shows characteristic cyclic voltammograms of the different La_1–*x*_Ca_*x*_MnO_3_ electrocatalysts supported on mesoporous carbon in an argon-saturated 0.1 M KOH solution at 0.010 V s^–1^. Whereas LaMnO_3_ features two cathodic reduction peaks located at 0.90 and 0.50 V, the voltammogram of CaMnO_3_ is characterized by a broad reduction peak centered at 0.80 V. The mixed compositions, La_0.4_Ca_0.6_MnO_3_ and La_0.6_Ca_0.4_MnO_3_, showed an intermediate behavior according to the major A-site component. The cathodic peak at ∼1.1 V is most intense for the high-Ca content samples, consistent with the systematic increase in redox current/charge with increasing Ca^2+^ substitution on the A-site. Higher Ca content increases the average Mn oxidation state, thereby increasing the number of Mn centers reduced/oxidized within the potential window and/or the magnitude of the Mn oxidation-state change per active site, leading to a larger cathodic Faradaic response. This behavior also aligns with the previously reported dependence of the electrochemical current on composition via surface B-site depletion, where the Ca-rich­(er) compositions can exhibit a higher density of accessible surface Mn redox sites.^24^ In our previous work, we showed that for LaMnO_3_, Mn^3+^ is formed, as the potential is made more negative, but Mn^4+^ ions are retained even at the most negative potentials achieved. However, CaMnO_3_ showed the formation of both Mn^3+^ and Mn^2+^, with little retention of Mn^4+^ at 0.2 V.^21^

**Figure 1:**
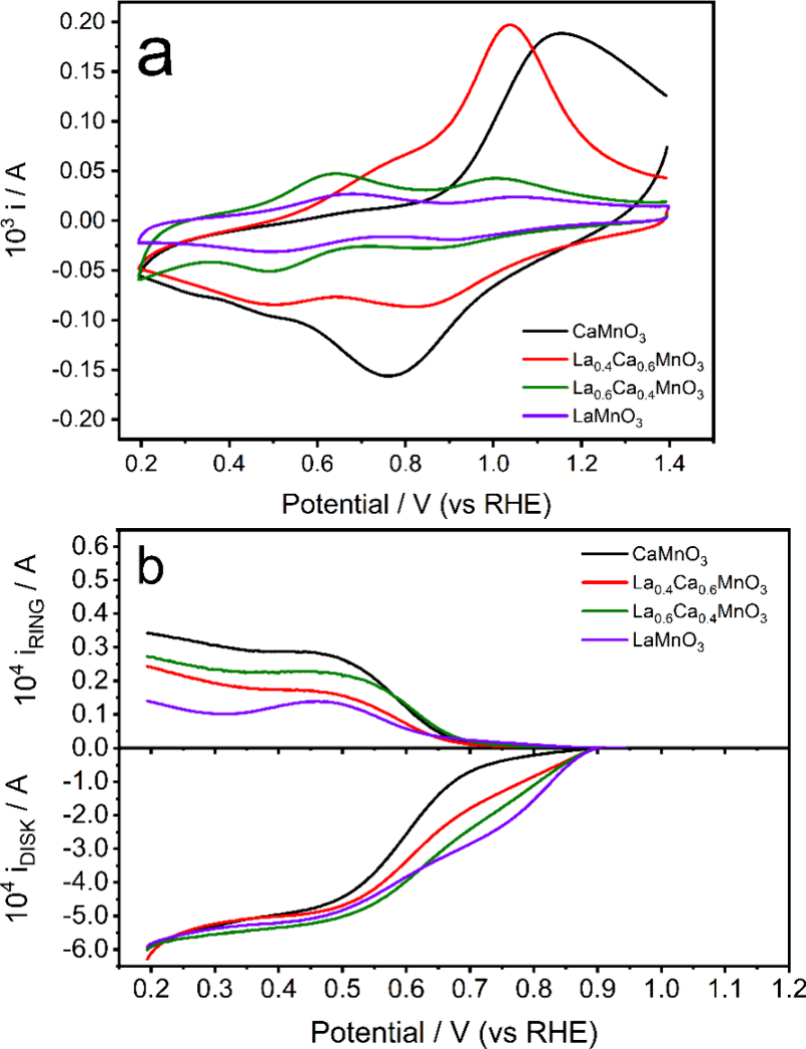
(a) Cyclic voltammograms of CaMnO_3_, La_0.4_Ca_0.6_MnO_3_, La_0.6_Ca_0.4_MnO_3_, and LaMnO_3_ nanoparticles in Ar-saturated 0.1 M KOH solution at 0.010 V s^–1^. (b) Rotating ring-disk electrode (RRDE) responses at 1600 rpm in O_2_-saturated 0.1 M KOH at 0.010 V s^–1^. The Pt ring was held at a constant potential of 1.10 V.

Figure 1b shows the disk (i_DISK_, bottom panel) and ring (i_RING_, top panel) currents obtained at 1600 rpm and 0.010 V s^–1^ in an O_2_-saturated 0.1 M KOH solution for all of the carbon-supported oxides. Substantial peroxide detection at the ring electrode is not observed until 0.7 V, indicating that ORR mainly occurs through a four-electron mechanism. The most positive onset potential, defined in this manuscript as the potential where the disk current reaches −50 μA, is observed for LaMnO_3_, while CaMnO_3_ requires a higher overpotential to initiate the reaction. As shown in previous works, the shift in ORR onset potential appears to mirror the displacement in the first peak of Mn reduction,^21,23,24^ consistent with linking the ORR kinetics with increasing electron density at the Mn site under operational conditions.

### In Situ XAS and XES Studies

HERFD-XANES data were collected, and the spectra are reported in Figure 2 for CaMnO_3_ (a), La_0.6_Ca_0.4_MnO_3_ (b), and LaMnO_3_ (c) (data for La_0.4_Ca_0.6_MnO_3_ is shown in Figure S1b). For all the samples, decreasing the potential triggers the reduction of Mn, as observed by the shift of both the edge and white-line positions to lower energy. Shifts of the edge position (defined as the half of the edge step) of 2.2, 2.0, 3.3, and 8.0 eV are observed between 1.40 and 0.20 V for LaMnO_3_, La_0.6_Ca_0.4_MnO_3_, La_0.4_Ca_0.6_MnO_3_, and CaMnO_3_, respectively. Although the Mn edge position is influenced by geometry and ligand field effects,^18,19^ making the estimation of Mn oxidation state from edge position somewhat approximate, the oxidation states of the La_*x*_Ca_1–*x*_MnO_3_ samples at various potentials determined by this method are presented in Figure S2 (HERFD-XANES spectra of the references used are presented in Figure S3). Croft et al. reported a method to estimate the average Mn oxidation state by looking at the position of the pre-edge peaks in the XANES region.^30^ The CaMnO_3_ pre-edge region is characterized by a single peak that shifts ∼2.5 eV between 1.40 and 0.2 V, whereas the position of the white line shifts by ∼6 eV. For LaMnO_3_, La_0.6_Ca_0.4_MnO_3_, and La_0.4_Ca_0.6_MnO_3_, the analysis of the pre-edge region is quite complex, as the spectra show two distinct peaks that change in intensity and position as the potential changes. HERFD-XANES suggests a stronger potential dependence of the Mn oxidation state for CaMnO_3_ than for the La-containing samples, in agreement with our earlier study.^21^

**Figure 2:**
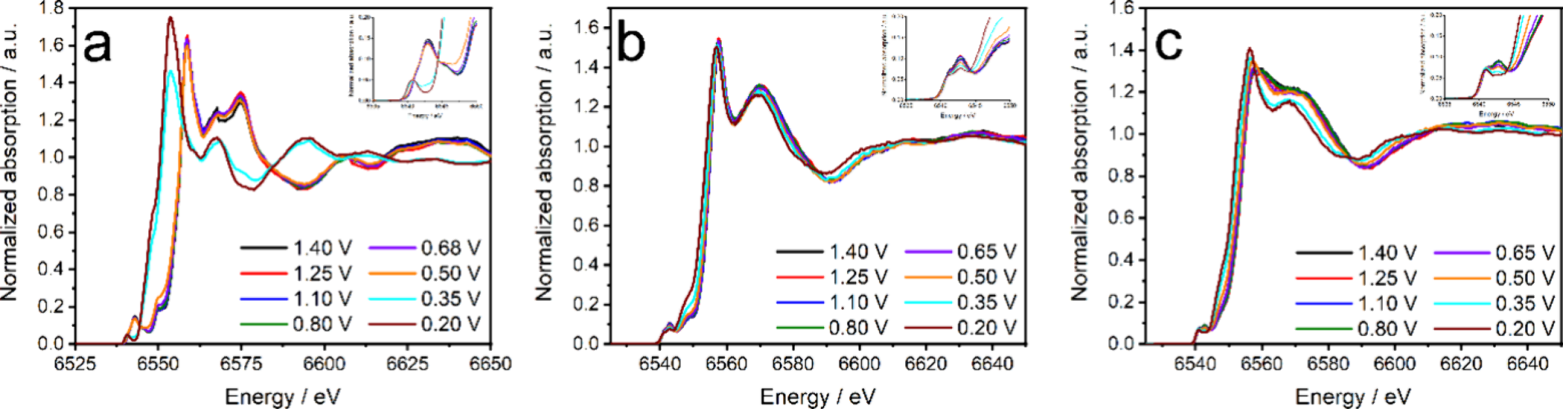
Normalized HERFD-XANES spectra obtained at the maximum of the Kβ_1,3_ line of CaMnO_3_ (a), La_0.6_Ca_0.4_MnO_3_ (b), and LaMnO_3_ (c). Insets show the corresponding pre-edge regions. Data were collected in N_2_-purged 0.1 M KOH.

In situ Kβ XES spectra were collected to further study the Mn valence changes over the potential range, taking advantage of the spin, oxidation state, and ligand sensitivity of the Mn Kβ lines.^31^ Figure S4 compares the Mn Kβ XES spectra of the La_1–*x*_Ca_*x*_MnO_3_ samples at different applied potentials, which feature the Kβ_1,3_ peak and a broad Kβ’ shoulder at lower energy. As the applied potential decreases, a decrease in the Mn valence is evidenced by a shift of the Kβ_1,3_ maximum to higher energy, accompanied by intensity changes in the Kβ′ region. To quantify changes in the oxidation state, the normalized Mn Kβ spectra collected for the reference samples were linearly combined to fit the Mn Kβ spectra of the La_1–*x*_Ca_*x*_MnO_3_ samples.^20,21,32^ Figure 3 shows the relative fractions of Mn­(II), Mn­(III), and Mn­(IV) species, calculated by linear combination of the XES standards (spectra for the references used are in Figure S5), as a function of the potential (representative fits of the XES spectra are shown in Figure S6 and statistics of the fits in Tables S1–S4) for CaMnO_3_ (a), La_0.6_Ca_0.4_MnO_3_ (b), and LaMnO_3_ (c) at different potentials during a cathodic scan (results for the anodic scan are shown in Figure S7 and results for La_0.4_Ca_0.6_MnO_3_ in Figure S8). For the cathodic scan, CaMnO_3_ (a) displays a strong dominance of Mn­(IV) at high potentials, which sharply transitions to Mn­(III) and Mn­(II) as the potential decreases. For La_0.6_Ca_0.4_MnO_3_ (b), the transition between Mn­(IV) and Mn­(III) is more gradual, with Mn­(III) dominating at intermediate potentials, and the fraction of Mn­(II) is constant over the potential range. LaMnO_3_ (c) exhibits a predominance of Mn­(IV) at high potentials, with Mn­(III) emerging as the major component at potentials lower than 0.6 V and Mn­(II) appearing only in a low percentage at the most negative potentials.

**Figure 3:**
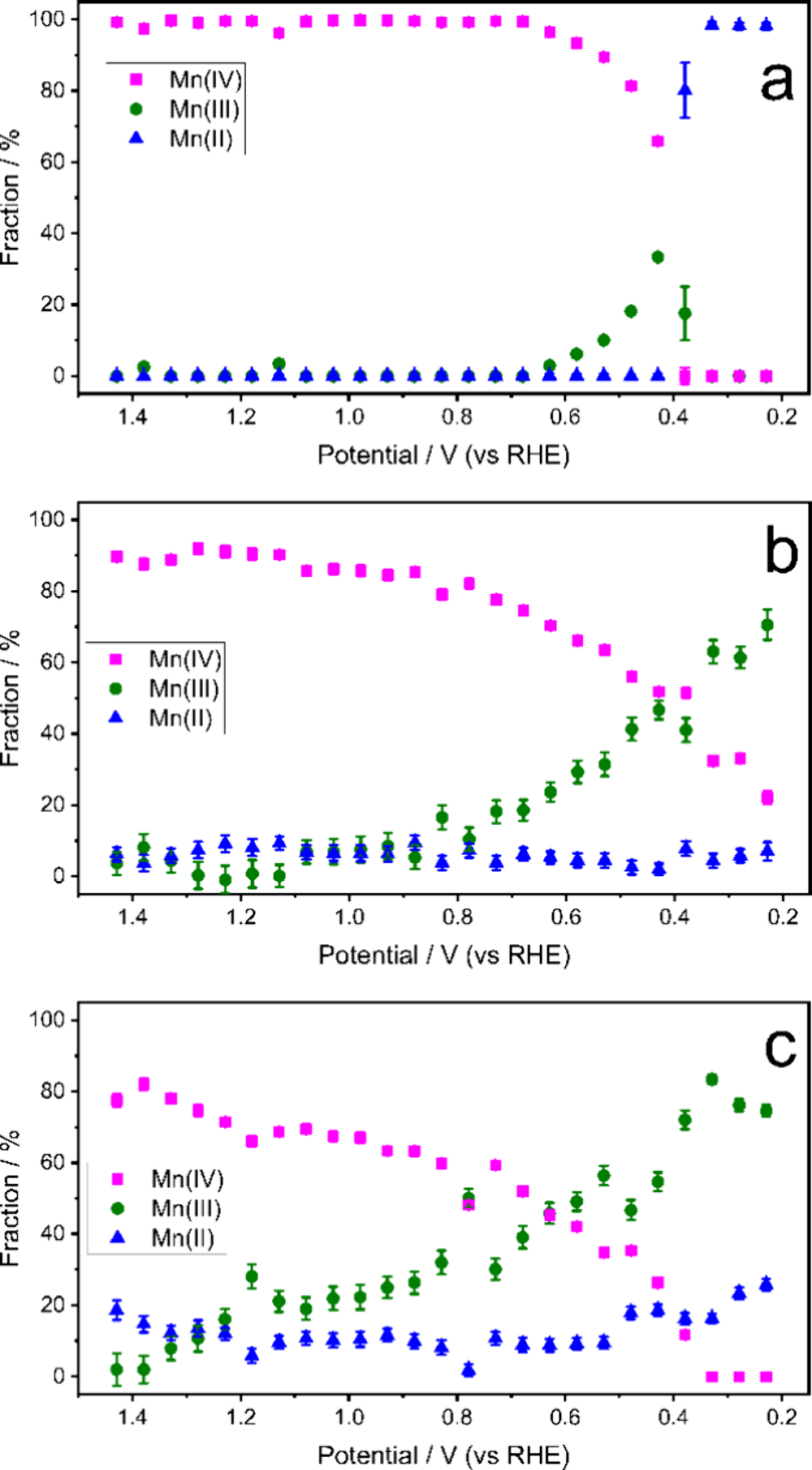
Percentage of the different cation species for CaMnO_3_ (a), La_0.6_Ca_0.4_MnO_3_ (b), and LaMnO_3_ (c) at different potentials in a cathodic scan obtained by linear combination fitting of the XES data.

Comparison of the cathodic and anodic scans reveals a hysteresis in the Mn oxidation-state transitions, particularly for CaMnO_3_, where the Mn­(IV)→Mn­(III) reduction occurs at significantly lower potentials (∼0.4 V) than the corresponding oxidation during the anodic scan (∼1.0 V). Nevertheless, the anodic scans (Figures S7 and S8) show that the initial Mn oxidation-state distribution is largely recovered upon reversal of the scan direction, indicating the overall reversibility of the redox process despite the shift in transition potentials. This hysteresis may be attributed to kinetic limitations and slow structural relaxation associated with Mn redox chemistry, including Jahn–Teller-related lattice distortions and oxygen vacancy formation and annihilation processes, which can lead to delayed equilibration during electrochemical cycling.^33–36^

Importantly, correlating spectroscopic signatures with electrochemical behavior shows that ORR onset coincides with the Mn­(IV) → Mn­(III) reduction. Peroxide-related features then emerge under more reducing conditions, becoming most pronounced when Mn­(II) formation is detectable (particularly for CaMnO_3_). This correspondence between Mn valence evolution and the electrochemical response supports a sequential 2e^–^ reduction to peroxide, followed by an additional 2e^–^ step that depends strongly on the composition. In the La-rich samples, peroxide formation is the most consistent with a kinetic limitation of the second 2e^–^ step, such that peroxide accumulates, and active sites are increasingly used to carry out the first reduction. In contrast, for Ca-rich samples, Mn­(IV) is not retained under operating conditions, and the presence of this species appears necessary to access the 4e^‑^ pathway; instead, the materials become over-reduced, and Mn­(II) is observed.

Figure 4 shows the Fourier-transformed Mn K-edge EXAFS spectra collected in standard fluorescence mode of CaMnO_3_ (a), La_0.6_Ca_0.4_MnO_3_ (b), and LaMnO_3_ (c) as a function of the applied electrode potential, providing insight into the structural dynamics (data for La_0.4_Ca_0.6_MnO_3_ are shown in Figure S9, and spectra in energy are shown in Figures S10 and S1a). LaMnO_3_ (Figure 4c) exhibits minimal changes with applied potential, suggesting that the Mn sites in LaMnO_3_ remain relatively unaffected by electrochemical cycling. In contrast, La_0.6_Ca_0.4_MnO_3_ (Figure 4b) exhibits a decrease in the amplitude of the Mn–O peak as the potential changes are made more negative, suggesting a decrease in Mn–O coordination. The most pronounced structural changes are observed in CaMnO_3_ (Figure 4a), where the spectra show substantial variations as the applied potential decreases. These changes suggest extensive Mn–O bond length expansion and reduction of the Mn–O coordination number.

**Figure 4:**
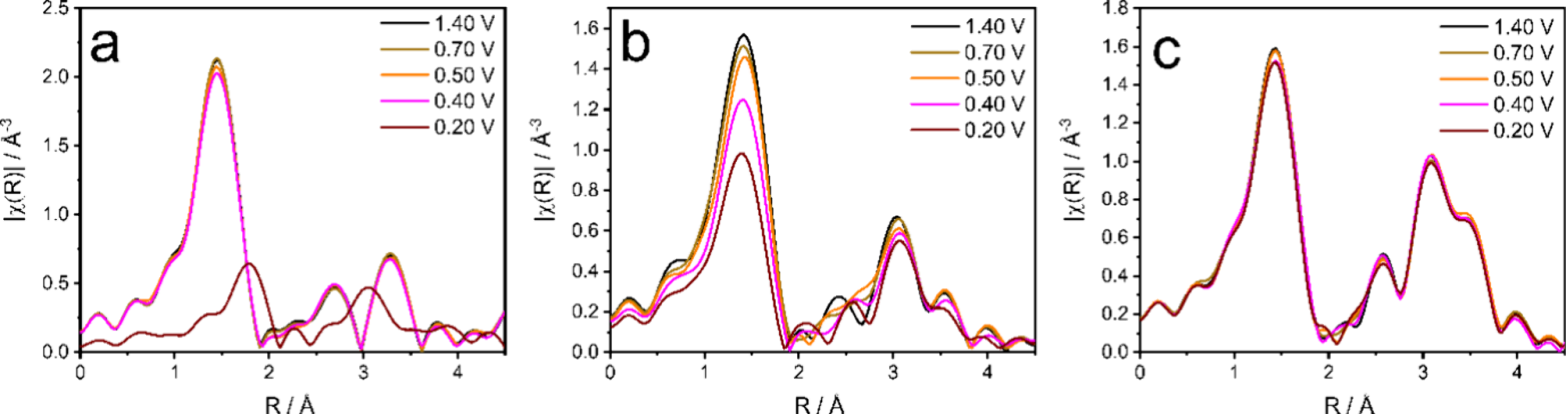
Fourier transforms of the k^2^-weighted Mn K-edge EXAFS of CaMnO_3_ (a), La_0.6_Ca_0.4_MnO_3_ (b), and LaMnO_3_ (c) at different potentials.

The perovskite structure was confirmed for the different materials at 1.40 V, as shown by the quality of the EXAFS fits in Figure S11 and Table S5. To determine the extent of the changes in structure suffered under potential conditions, first coordination shell fits of the EXAFS data were performed (results shown in Table S6). LaMnO_3_ shows minimal changes in Mn–O bond length, coordination number, and disorder across all applied potentials. In contrast, CaMnO_3_ undergoes substantial structural modifications with increasing Mn–O disorder and deviations in coordination number, indicating a loss of the perovskite framework under operational conditions. Mn leaching appears unlikely over the time frame of the XAS measurements (approximately 1 h per potential hold) because the Mn Kα fluorescence intensity remained unchanged within experimental uncertainty. However, since La/Ca edges were not measured, partial A-site (La/Ca) dissolution cannot be ruled out as a contributor to the observed phase instability and reconstruction. La_0.6_Ca_0.4_MnO_3_ and La_0.4_Ca_0.6_MnO_3_ exhibit moderate structural evolution, with noticeable changes in Mn coordination and bond length, but they retain their perovskite structures while adapting to redox cycling. Figure S12 shows the Fourier-transformed Mn K-edge EXAFS spectra of the synthesized materials at the initial 1.4 V condition and at 1.4 V after cycling. The structural changes appear to be reversible for LaMnO_3_ and La_0.6_Ca_0.4_MnO_3_. While the first coordination shell seems to be preserved in La_0.4_Ca_0.6_MnO_3_, changes are observed in the second shell. In contrast, CaMnO_3_ undergoes irreversible structural changes due to collapse of the perovskite framework.

XAS and XES provide a quantitative analysis of the changes in oxidation state of Mn sites operating in the potential range associated with the ORR. The evolution of the spectral responses is described in terms of mixed oxidation states, which represent variation of the electron population of Mn 3*d* states distributed in the range of 0.4–1.3 V vs RHE. In a previous study, the density of these states in LaMn_*x*_Ni_1–*x*_O_3_ was quantified using pseudocapacitance responses (similar to those shown in Figure 1), together with DFT calculations, showing that the ORR kinetics exhibits a second-order dependence on the density of electrons stored in the Mn 3*d* states.^37^ In the present La_*x*_Ca_1–*x*_MnO_3_ series, however, Ca-rich compositions display slower ORR kinetics than La-rich compositions, despite their substantially larger pseudocapacitive charge. As investigated previously, this preparation method yields ∼30 nm, phase-pure rhombohedral LaMnO_3_ (R-3cH space group), while the introduction of Ca^2+^ promotes the orthorhombic Pnma space group.^24^ This observation suggests a higher intrinsic activity of Mn sites in the rhombohedral space group, while the stability of the orthorhombic phase is compromised upon populating Mn sites under the ORR-relevant conditions.

## Conclusions

This work highlights the effectiveness of both XAS and XES for disentangling the electronic and structural dynamics of Mn-based perovskite oxides under oxygen reduction conditions. By using both Mn K-edge EXAFS and Kβ XES analyses, albeit in separate experiments, we were able to monitor changes in both the oxidation state and coordination environment of Mn ions during electrochemical operation.

Our results reveal that structural and redox responses are strongly composition-dependent: LaMnO_3_ remains largely unchanged, CaMnO_3_ undergoes irreversible structural degradation according to the EXAFS fitting, which is accompanied by Mn­(II) formation, as evidenced clearly in both the edge-shift in the conventional Kα XAS and the detailed analysis of the Kβ XES, while La_1–*x*_Ca_*x*_MnO_3_ compositions show reversible Mn­(IV)/Mn­(III) redox activity, as highlighted in the XES analysis. Importantly, the additional information provided by XES enables the contributions of each Mn species to the oxygen reduction mechanism to be resolved in greater detail, providing direct insight into how specific redox states correlate with (in)­stability. In particular, XES provides unique confirmation that significant formation of Mn­(II) emerges only in CaMnO_3_, directly linking over-reduction to structural instability.

Overall, this integrated XAS-XES approach enables a direct connection among redox chemistry, structure, and catalytic response, providing a powerful framework for designing Mn-based oxides with improved activity and stability.

## Supplementary Material



## Notes

Supplementary X-ray spectroscopy data and analysis, including Mn K-edge (HERFD-)­XANES and Kβ XES spectra, EXAFS Fourier transforms and fitting results, and linear-combination/structural analysis tables (PDF)
