# Supplementary material for: Detailed Understanding of the Electrochemistry and Oxygen Reduction Activity of La1–x Ca x MnO3 Obtained by In Situ XAS and XES
Source: ACS Electrochem. 2026 Apr 14;2(5):1190–6. doi: 10.1021/acselectrochem.5c00541 (PMC13158912; doi:10.1021/acselectrochem.5c00541)
Supplement: Supplementary file 1 [file ec5c00541_si_001.pdf]

Detailed understanding of the electrochemistry and oxygen reduction activity of $La_{1-x}Ca_xMnO_3$ obtained by *in situ* XAS and XES

Veronica Celorrio,[1]* Haoliang Huang[2, 3], Shusaku Hayama[1], David J. Fermin[4], Andrea E. Russell[2]*

[1]Diamond Light Source Ltd. Diamond House. Harwell Campus. Didcot, OX11 0DE, UK.

[2]School of Chemistry and Chemical Engineering, University of Southampton. Highfield, Southampton, SO17 1BJ UK.

[3]Songshan Lake Materials Laboratory, Dongguan 523808, China

[4]School of Chemistry, University of Bristol, Cantocks Close, Bristol BS8 1TS, UK.

Table of contents.

Figure S1. *XANES spectra at the Mn K-edge (a) HERFD-XANES spectra obtained at the maximum of the $K\beta_{1,3}$ line (b) for $La_{0.4}Ca_{0.6}MnO_3$ recorded at different applied potentials. Inset shows the corresponding pre-edge regions. Data collected in N_2-purged 0.1 M KOH*………S1

Figure S2. *Calibration plot for the determination of the average Mn oxidation state from the edge positions of the HERFD-XANES data of the $CaMnO_3$ (a), $La_{0.6}Ca_{0.4}MnO_3$ (b), $La_{0.4}Ca_{0.6}MnO_3$ (c) and $LaMnO_3$ (d) electrodes as a function of the potential*………………….S2

Figure S3. *Normalised HERFD-XANES spectra obtained at the maximum of the $K\beta_{1,3}$ line of MnO_2, Mn_2O_3 and MnO oxides used as references*…………………………………………………......S3

Figure S4. *Normalised Mn Kβ XES spectra for $CaMnO_3$ (a), $La_{0.4}Ca_{0.6}MnO_3$ (b), $La_{0.6}Ca_{0.4}MnO_3$ and $LaMnO_3$ (d) at different applied potentials*…………………………………S4

Figure S5. *Normalised Mn Kβ XES spectra for MnO_2, Mn_2O_3 and MnO references*…………..S5

Figure S6. *Linear combination of the normalized Mn Kβ XES spectra for $CaMnO_3$ (a), $La_{0.6}Ca_{0.4}MnO_3$ (b), $La_{0.4}Ca_{0.6}MnO_3$ (c) and $LaMnO_3$ (d) electrodes at 0.58 V*………….……S6

Figure S7. *Percentage of the different cation species for $CaMnO_3$ (a), $La_{0.6}Ca_{0.4}MnO_3$ (b) and $LaMnO_3$ (c) at different potentials in an anodic scan obtained by linear combination fitting of the XES data*……………………………………………………………………………………S7

Figure S8. *Percentage of the different cation species for $La_{0.4}Ca_{0.6}MnO_3$ at different potentials in the cathodic (a) and anodic (b) scan obtained by linear combination fitting of the XES data*……………………………………………………………………………………………S8

Figure S9. *Fourier transforms of the k^2-weighted Mn K-edge EXAFS of $La_{0.4}Ca_{0.6}MnO_3$ at different potentials* …………………………………………………………………………….....S9

Figure S10. *XANES spectra at the Mn K-edge for $CaMnO_3$ (a), $La_{0.6}Ca_{0.4}MnO_3$ (b) and $LaMnO_3$ (c) perovskite electrodes recorded at different applied potentials* …………………....S10

Figure S11. *Data (black line) and fits (red line) of the k^2-weighted EXAFS signals in k-space for $CaMnO_3$ (a), $La_{0.4}Ca_{0.6}MnO_3$ (c), $La_{0.6}Ca_{0.4}MnO_3$ (e) and $LaMnO_3$ (g) at OCP. Data (black lines) and fits (red lines) of the magnitude of FT signal of the k^2-weighted EXAFS for $CaMnO_3$ (b), $La_{0.4}Ca_{0.6}MnO_3$ (d), $La_{0.6}Ca_{0.4}MnO_3$ (f) and $LaMnO_3$ (h) at OCP. $LaMnO_3$: $2.6 < k < 11.0$ Å^{-1}; $1.1 < R < 4.0$ Å. $CaMnO_3$: $3.0 < k < 12.0$ Å^{-1}; $1.0 < R < 4.0$ Å^{-1}*…………………………….S11

Figure S12. *Fourier transforms of the k^2-weighted Mn K-edge EXAFS of $CaMnO_3$ (a), $La_{0.4}Ca_{0.6}MnO_3$ (b), $La_{0.6}Ca_{0.4}MnO_3$ (c) and $LaMnO_3$ (d) at 1.4 V before and after electrochemical cycling*…………………………………………………………………………....S12

Table S1. *Results of the linear combination analysis of $CaMnO_3$ XES spectra by the references MnO_2, Mn_2O_3 and MnO at different potentials in a cathodic (blue) and anodic (yellow) scan*…………………………………………………………………………………………….S13

Table S2. *Results of the linear combination analysis of $La_{0.4}Ca_{0.6}MnO_3$ XES spectra by the references MnO_2, Mn_2O_3 and MnO at different potentials in a cathodic (blue) and anodic (yellow) scan*…………………………………………………………………………………....S14

Table S3. *Results of the linear combination analysis of $La_{0.6}Ca_{0.4}MnO_3$ XES spectra by the references MnO_2, Mn_2O_3 and MnO at different potentials in a cathodic (blue) and anodic (yellow) scan*…………………………………………………………………………………....S15

Table S4. *Results of the linear combination analysis of $LaMnO_3$ XES spectra by the references MnO_2, Mn_2O_3 and MnO at different potentials in a cathodic (blue) and anodic (yellow) scan*…………………………………………………………………………………………….S16

Table S5. *Relative energy shift and the best fit results from the structural analysis of $La_{1-x}Ca_xMnO_3$ at the Mn K-edge and at OCP. N is the coordination number, R is the interatomic distance Mn-O and σ^2 is the Debye-Waller factor. R_f is the R-factor, which represents the relative error of the fit and data. $CaMnO_3$: $2.7 < k < 12.2$; $1.0 < R < 4.0$. $La_{0.4}Ca_{0.6}MnO_3$: $2.8 < k < 11.1$; $1.0 < R < 4.0$; $La_{0.6}Ca_{0.4}MnO_3$: $2.8 < k < 11.0$; $1.0 < R < 4.0$; $LaMnO_3$: $2.6 < k < 11.1$; $1.0 < R < 4.0$*………………………………………………………….S17

Table S6. *Best fit results from the structural analysis of the first coordination shell of $La_{1-x}Ca_xMnO_3$ at the Mn K-edge and at different potential conditions. N is the coordination number, R is the interatomic distance Mn-O and σ^2 is the Debye-Waller factor. R_f is the R-factor, which represents the relative error of the fit and data*……………………………………....S18

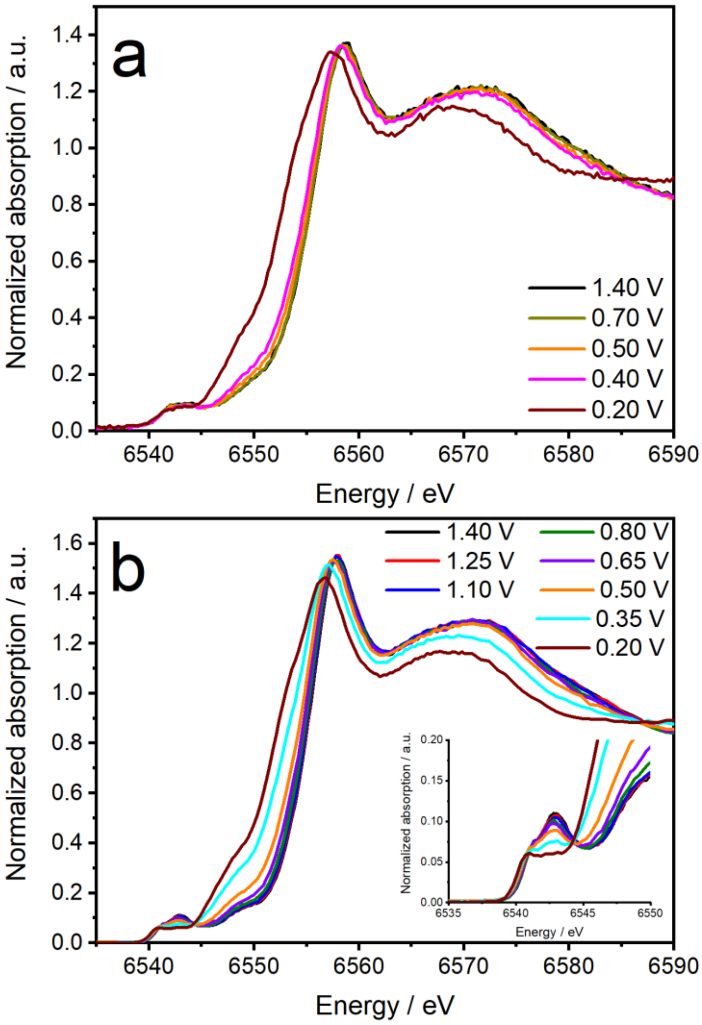

Figure S1. XANES spectra at the Mn K-edge (a) HERFD-XANES spectra obtained at the maximum of the K$\beta_{1,3}$ line (b) for $La_{0.4}Ca_{0.6}MnO_3$ recorded at different applied potentials. Inset shows the corresponding pre-edge regions. Data collected in N_2-purged 0.1 M KOH.

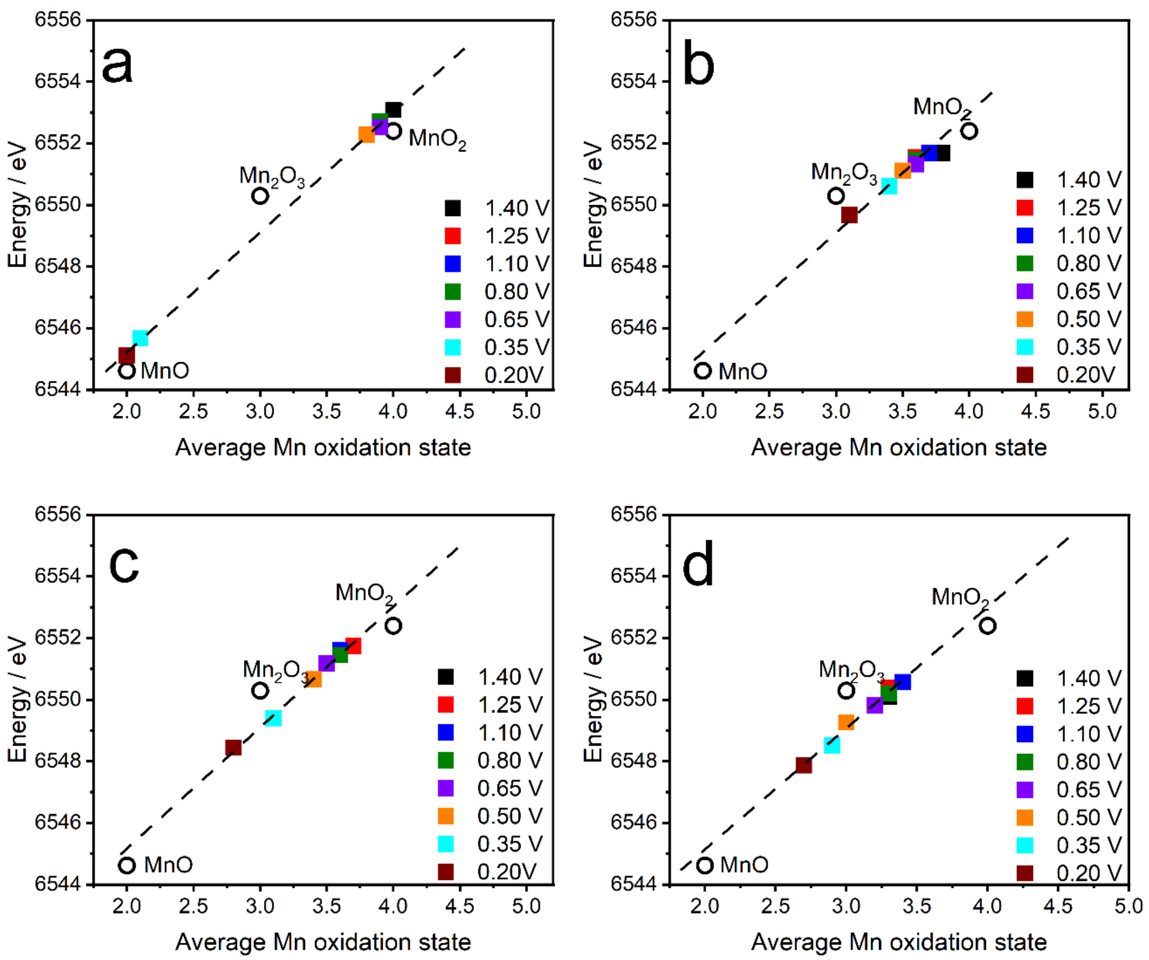

Figure S2. Calibration plot for the determination of the average Mn oxidation state from the edge positions of the HERFD-XANES data of the $CaMnO_3$ (a), $La_{0.6}Ca_{0.4}MnO_3$ (b), $La_{0.4}Ca_{0.6}MnO_3$ (c) and $LaMnO_3$ (d) electrodes as a function of the potential.

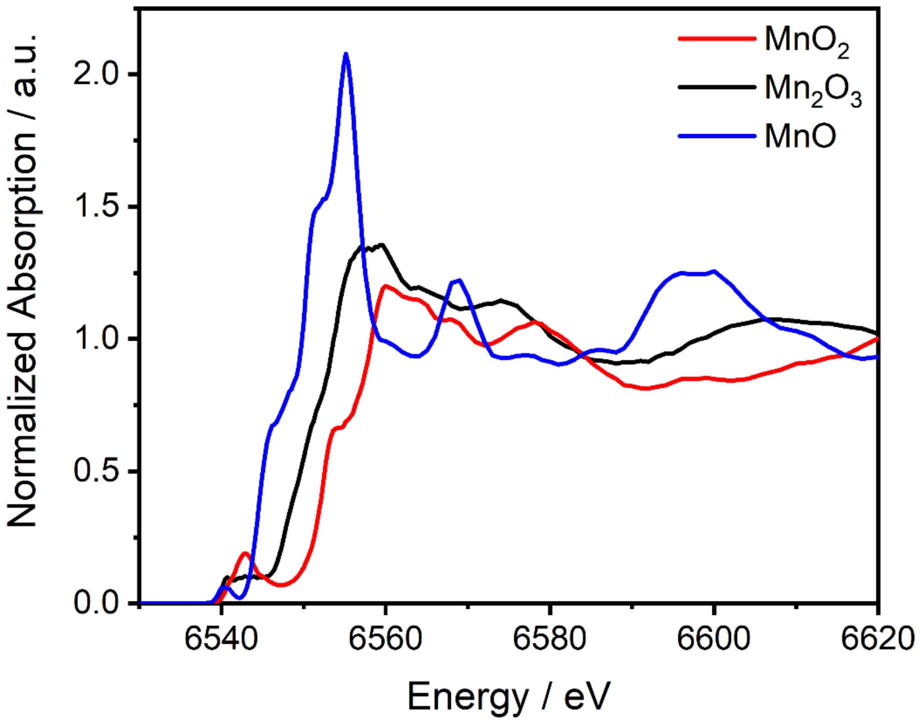

Figure S3. Normalised HERFD-XANES spectra obtained at the maximum of the K$\beta_{1,3}$ line of MnO_2, Mn_2O_3 and MnO oxides used as references.

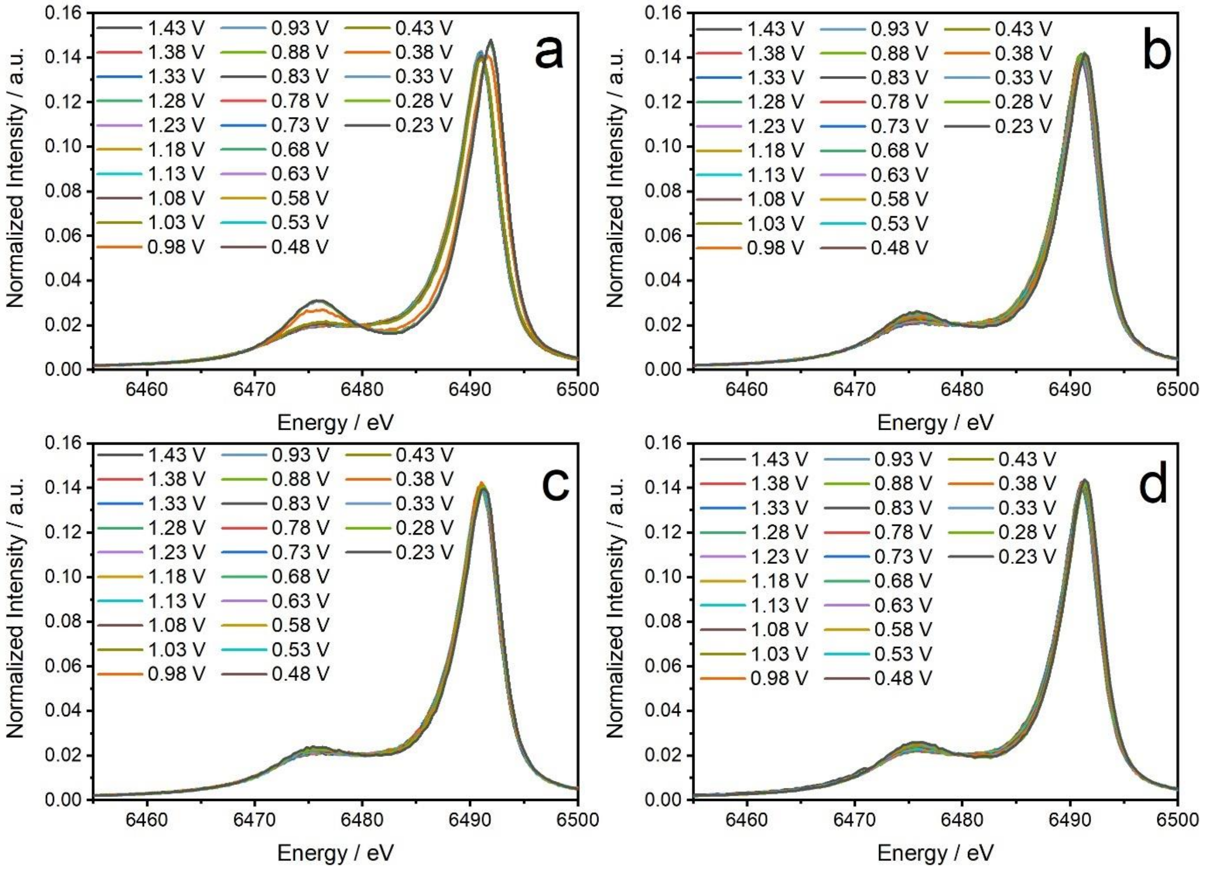

Figure S4. Normalised Mn Kβ XES spectra for $CaMnO_3$ (a), $La_{0.4}Ca_{0.6}MnO_3$ (b), $La_{0.6}Ca_{0.4}MnO_3$ and $LaMnO_3$ (d) at different applied potentials.

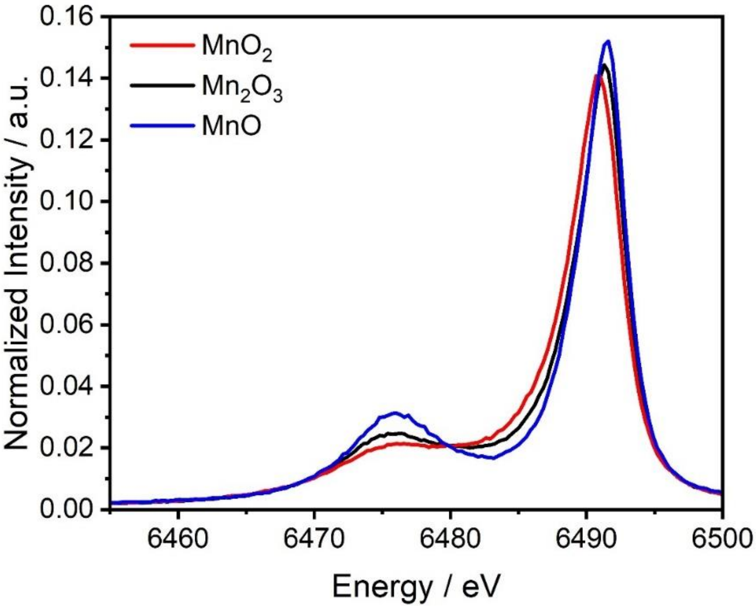

Figure S5. Normalised Mn Kβ XES spectra for MnO_2, Mn_2O_3 and MnO references.

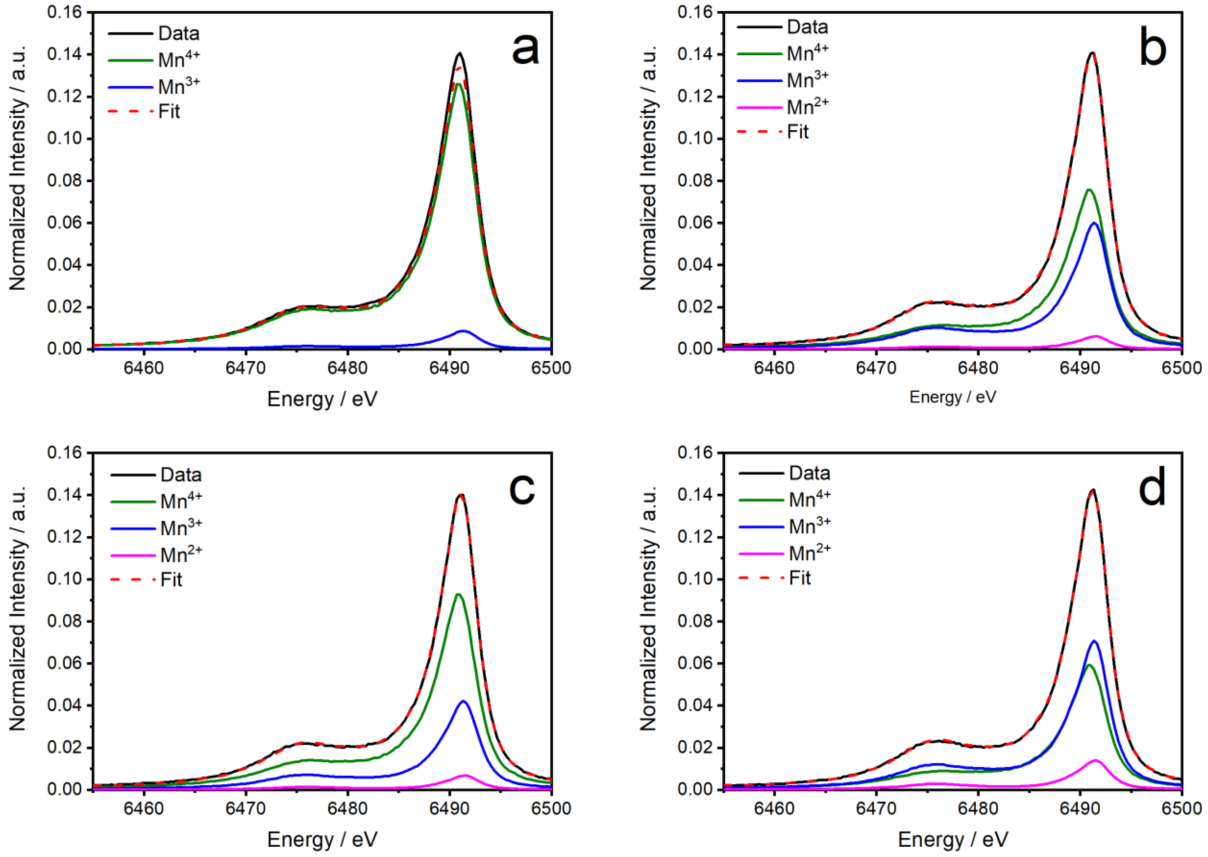

Figure S6. Linear combination of the normalized Mn Kβ XES spectra for CaMnO3 (a), $La_{0.6}Ca_{0.4}MnO_3$ (b), $La_{0.4}Ca_{0.6}MnO_3$ (c) and $LaMnO_3$ (d) electrodes at 0.58 V.

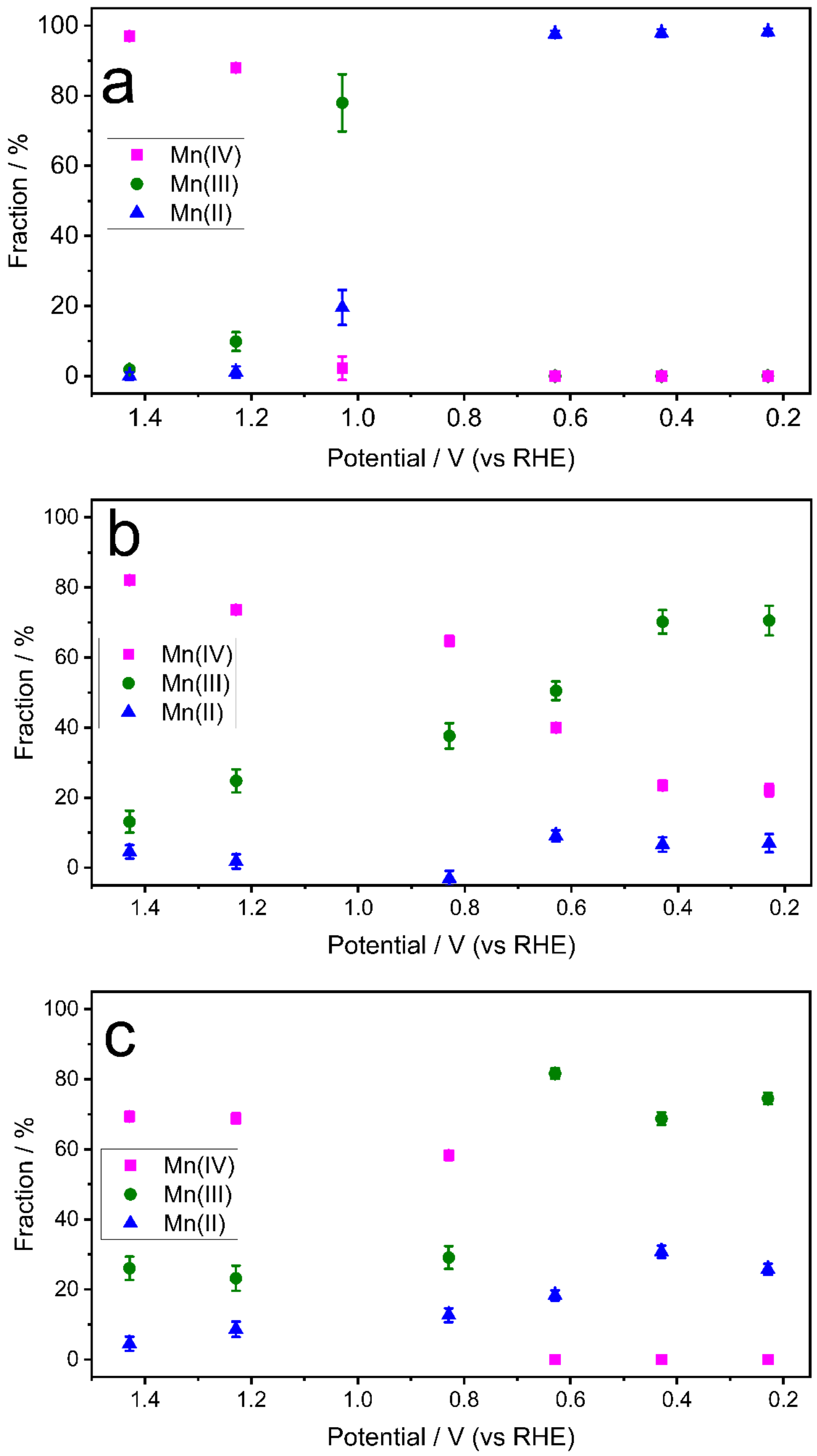

Figure S7. Percentage of the different cation species for $CaMnO_3$ (a), $La_{0.6}Ca_{0.4}MnO_3$ (b) and $LaMnO_3$ (c) at different potentials in an anodic scan obtained by linear combination fitting of the XES data.

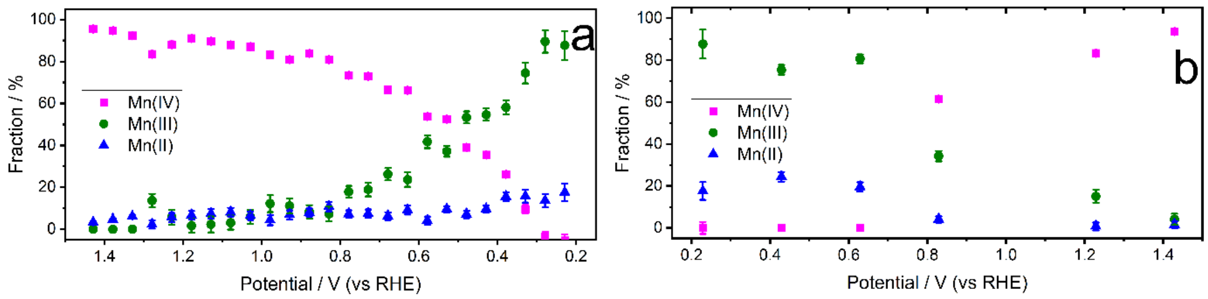

Figure S8. Percentage of the different cation species for $La_{0.4}Ca_{0.6}MnO_3$ at different potentials in the cathodic (a) and anodic (b) scan obtained by linear combination fitting of the XES data.

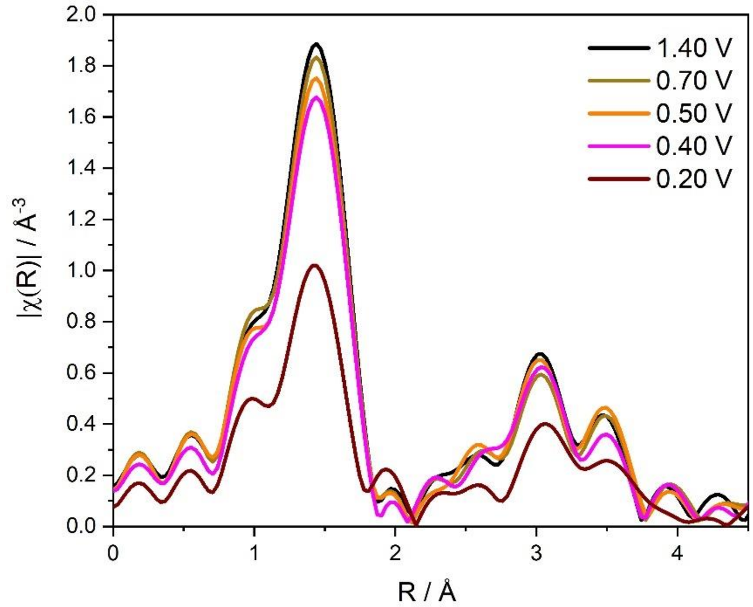

Figure S9. Fourier transforms of the k^2-weighted Mn K-edge EXAFS of $La_{0.4}Ca_{0.6}MnO_3$ at different potentials.

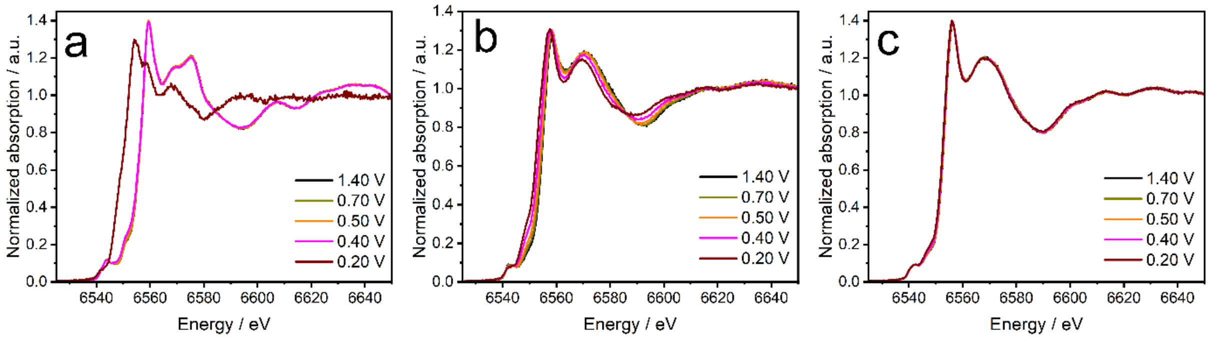

Figure S10. XANES spectra at the Mn K-edge for $CaMnO_3$ (a), $La_{0.6}Ca_{0.4}MnO_3$ (b) and $LaMnO_3$ (c) perovskite electrodes recorded at different applied potentials.

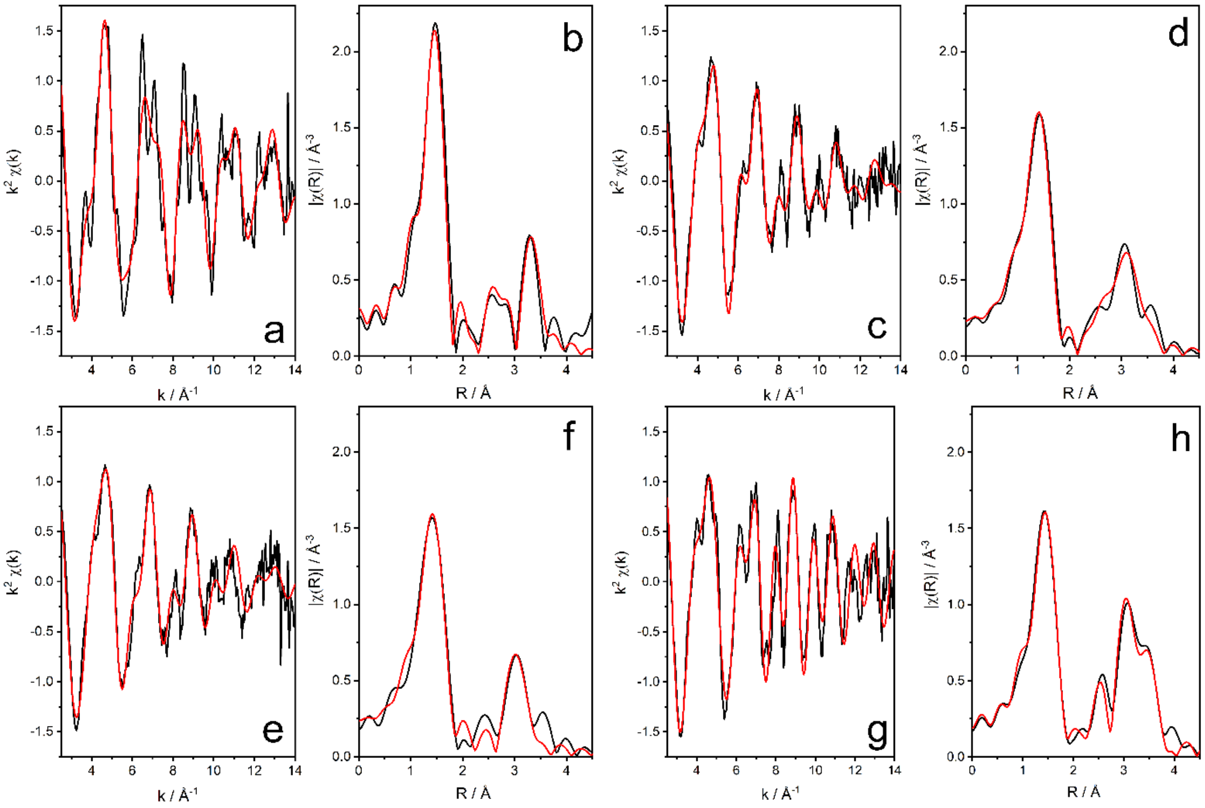

Figure S11. Data (black line) and fits (red line) of the k^2-weighted EXAFS signals in k-space for $CaMnO_3$ (a), $La_{0.4}Ca_{0.6}MnO_3$ (c), $La_{0.6}Ca_{0.4}MnO_3$ (e) and $LaMnO_3$ (g) at OCP. Data (black lines) and fits (red lines) of the magnitude of FT signal of the k^2-weighted EXAFS for $CaMnO_3$ (b), $La_{0.4}Ca_{0.6}MnO_3$ (d), $La_{0.6}Ca_{0.4}MnO_3$ (f) and $LaMnO_3$ (h) at OCP. $LaMnO_3$: $2.6 < k < 11.0$ $Å^{-1}$; $1.1 < R < 4.0$ Å. $CaMnO_3$: $3.0 < k < 12.0$ $Å^{-1}$; $1.0 < R < 4.0$ $Å^{-1}$.

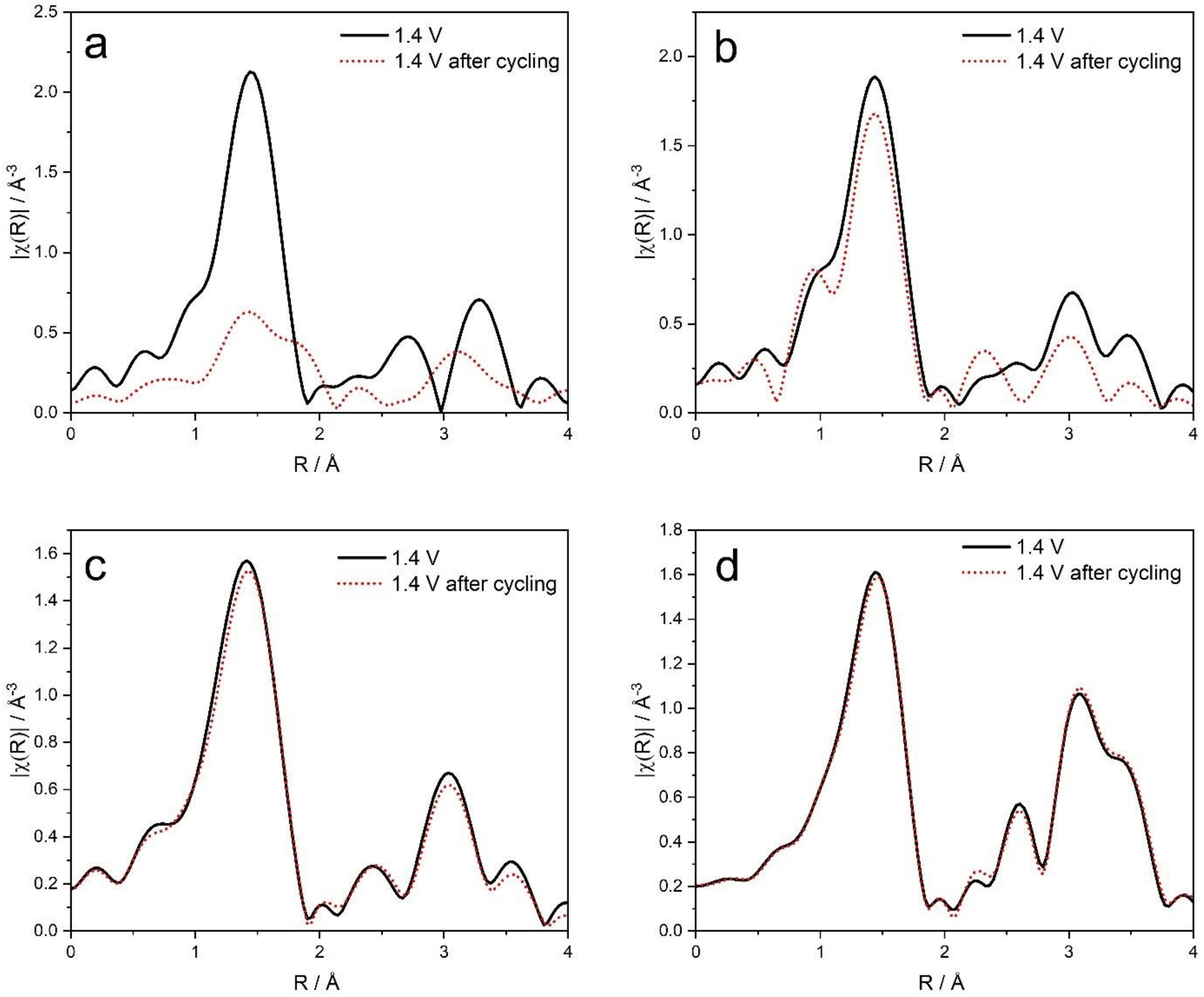

Figure S12. Fourier transforms of the k^2-weighted Mn K-edge EXAFS of $CaMnO_3$ (a), $La_{0.4}Ca_{0.6}MnO_3$ (b), $La_{0.6}Ca_{0.4}MnO_3$ (c) and $LaMnO_3$ (d) at 1.4 V before and after electrochemical cycling.

Table S1. Results of the linear combination analysis of $CaMnO_3$ XES spectra by the references MnO_2, Mn_2O_3 and MnO at different potentials in a cathodic (blue) and anodic (yellow) scan.

Cathodic scan						Anodic scan					
Potential / V	% Mn^{4+}	% Mn^{3+}	% Mn^{2+}	Average Mn oxidation state	Adj. R-Square	Potential / V	% Mn^{4+}	% Mn^{3+}	% Mn^{2+}	Average Mn oxidation state	Adj. R-Square
1.43	99.2 ± 0.2	0.0 ± 0.4	0.0 ± 0.4	4.0 ± 0.0	0.9929	0.58	89.5 ± 0.8	6.1 ± 0.8	0.0 ± 0.5	3.9 ± 0.1	0.9931
1.38	97.5 ± 1.1	2.5 ± 1.2	0.0 ± 0.0	4.0 ± 0.1	0.9929	0.53	81.5 ± 1.0	10.0 ± 0.9	0.0 ± 0.5	3.9 ± 0.1	0.9931
1.33	97.7 ± 0.2	0.0 ± 0.1	0.0 ± 0.1	4.0 ± 0.0	0.9929	0.48	65.9 ± 0.7	18.2 ± 1.0	0.0 ± 0.3	3.8 ± 0.1	0.9930
1.28	99.1 ± 0.1	0.0 ± 0.5	0.0 ± 0.5	4.0 ± 0.0	0.9930	0.43	0.0 ± 2.3	33.4 ± 0.7	0.0 ± 0.7	3.6 ± 0.1	0.9931
1.23	99.5 ± 1.2	0.0 ± 0.0	0.0 ± 0.0	4.0 ± 0.1	0.9929	0.38	0.0 ± 0.8	17.5 ± 7.5	80.2 ± 7.7	2.1 ± 0.7	0.9872
1.18	99.5 ± 0.1	0.0 ± 0.2	0.0 ± 0.2	4.0 ± 0.0	0.9931	0.33	0.0 ± 0.9	0.0 ± 0.8	98.5 ± 0.9	2.0 ± 0.1	0.9803
1.13	96.2 ± 0.1	3.4 ± 1.1	0.0 ± 0.4	4.0 ± 0.1	0.9930	0.28	0.0 ± 0.9	0.0 ± 0.9	98.2 ± 1.0	2.0 ± 0.1	0.9791
1.08	99.5 ± 0.1	0.0 ± 0.5	0.0 ± 0.5	4.0 ± 0.0	0.9930	0.23	0.0 ± 1.1	0.0 ± 0.9	98.2 ± 1.0	2.0 ± 0.1	0.9781
1.03	99.7 ± 0.2	0.0 ± 0.3	0.0 ± 0.3	4.0 ± 0.0	0.9929	0.43	0.0 ± 1.1	0.0 ± 1.1	97.9 ± 1.1	2.0 ± 0.1	0.9748
0.98	99.8 ± 0.2	0.0 ± 0.2	0.0 ± 0.2	4.0 ± 0.0	0.9929	0.63	0.0 ± 1.2	0.0 ± 1.2	97.6 ± 1.0	2.0 ± 0.1	0.9777
0.93	99.7 ± 0.1	0.0 ± 0.3	0.0 ± 0.3	4.0 ± 0.0	0.9929	1.03	2.2 ± 3.3	78.0 ± 8.2	19.5 ± 5.0	2.8 ± 0.7	0.9924
0.88	99.2 ± 0.1	0.0 ± 0.3	0.0 ± 0.3	4.0 ± 0.0	0.9930	1.23	88.0 ± 1.1	9.8 ± 2.7	1.1 ± 1.6	3.8 ± 0.2	0.9931
0.83	99.3 ± 0.1	0.0 ± 0.8	0.0 ± 0.8	4.0 ± 0.1	0.9930	1.43	97.0 ± 0.7	1.9 ± 0.7	0.0 ± 1.1	3.9 ± 0.1	0.9931
0.78	99.6 ± 0.1	0.0 ± 0.7	0.0 ± 0.7	4.0 ± 0.1	0.9930						
0.73	99.5 ± 0.1	0.0 ± 0.4	0.0 ± 0.4	4.0 ± 0.0	0.9931						
0.68	96.5 ± 1.0	0.0 ± 0.5	0.0 ± 0.5	4.0 ± 0.0	0.9930						
0.63	93.4 ± 0.8	2.9 ± 1.0	0.0 ± 0.6	3.9 ± 0.1	0.9930						

Table S2. Results of the linear combination analysis of $La_{0.4}Ca_{0.6}MnO_3$ XES spectra by the references MnO_2, Mn_2O_3 and MnO at different potentials in a cathodic (blue) and anodic (yellow) scan.

Cathodic scan						Anodic scan					
Potential / V	% Mn^{4+}	% Mn^{3+}	% Mn^{2+}	Average Mn oxidation state	Adj. R-Square	Potential / V	% Mn^{4+}	% Mn^{3+}	% Mn^{2+}	Average Mn oxidation state	Adj. R-Square
1.43	95.6 ± 0.5	0.0 ± 0.0	3.3 ± 0.4	3.9 ± 0.0	0.9931	0.58	53.8 ± 1.3	41.8 ± 3.1	4.1 ± 1.9	3.5 ± 0.3	0.9931
1.38	94.8 ± 0.5	0.0 ± 0.0	4.6 ± 0.5	3.9 ± 0.0	0.9931	0.53	52.6 ± 1.0	37.1 ± 2.5	9.5 ± 1.6	3.4 ± 0.2	0.9931
1.33	92.4 ± 0.5	0.0 ± 0.0	6.2 ± 0.5	3.8 ± 0.0	0.9931	0.48	39.0 ± 1.2	53.3 ± 2.9	7.0 ± 1.7	3.3 ± 0.2	0.9931
1.28	83.6 ± 1.3	13.6 ± 3.2	2.2 ± 1.9	3.8 ± 0.3	0.9931	0.43	35.6 ± 1.2	54.7 ± 2.9	9.6 ± 1.8	3.3 ± 0.2	0.9931
1.23	88.1 ± 1.3	5.7 ± 3.6	5.6 ± 2.2	3.8 ± 0.3	0.9931	0.38	26.3 ±1.4	58.2 ± 3.3	15.4 ± 2.0	3.1 ± 0.3	0.9931
1.18	91.1 ± 1.3	1.7 ± 3.3	6.7 ± 2.0	3.8 ± 0.3	0.9931	0.33	9.5 ± 2.0	74.5 ± 4.9	15.7 ± 3.0	2.9 ± 0.4	0.9929
1.13	89.6 ± 1.5	2.3 ± 3.7	7.4 ± 2.3	3.8 ± 0.3	0.9930	0.28	-3.4 ± 2.2	89.5 ± 5.3	13.6 ± 3.2	2.8 ± 0.4	0.9929
1.08	87.9 ± 1.4	3.1 ± 3.3	8.1 ± 2.0	3.8 ± 0.3	0.9931	0.23	0.0 ± 2.8	87.7 ± 6.9	17.6 ± 4.2	2.8 ± 0.6	0.9927
1.03	87.1 ± 1.4	5.9 ± 3.3	6.4 ± 2.0	3.8 ± 0.3	0.9931	0.43	0.0 ± 0.0	75.4 ± 2.3	24.2 ± 2.3	2.7 ± 0.2	0.9927
0.98	83.3 ± 1.6	12.2 ± 4.0	4.3 ± 2.4	3.8 ± 0.3	0.9930	0.63	0.0 ± 0.0	80.6 ± 2.1	19.5 ± 2.2	2.8 ± 0.2	0.9928
0.93	81.0 ± 1.4	11.1 ± 3.5	6.9 ± 2.1	3.7 ± 0.3	0.9931	0.83	61.5 ± 1.0	34.2 ± 2.5	4.0 ± 1.5	3.6 ± 0.2	0.9931
0.88	83.9 ± 1.3	8.2 ± 3.2	8.0 ± 1.9	3.8 ± 0.3	0.9931	1.23	83.4 ± 1.3	15.1 ± 3.1	0.7 ± 1.9	3.8 ± 0.3	0.9931
0.83	80.9 ± 1.5	7.3 ± 3.6	10.8 ± 2.2	3.7 ± 0.3	0.9931	1.43	93.8 ± 1.1	4.1 ± 2.7	1.5 ± 1.6	3.9 ± 0.2	0.9931
0.78	73.5 ± 1.2	17.9 ± 2.8	7.2 ± 1.7	3.6 ± 0.2	0.9931						
0.73	72.9 ± 1.4	18.9 ± 3.3	7.4 ± 2.0	3.6 ± 0.3	0.9931						
0.68	66.5 ± 1.2	26.3 ± 3.0	6.3 ± 1.8	3.6 ± 0.2	0.9931						
0.63	66.3 ± 1.4	23.7 ± 3.4	9.1 ± 2.1	3.5 ± 0.3	0.9931						

Table S3. Results of the linear combination analysis of $La_{0.6}Ca_{0.4}MnO_3$ XES spectra by the references MnO_2, Mn_2O_3 and MnO at different potentials in a cathodic (blue) and anodic (yellow) scan.

Cathodic scan						Anodic scan					
Potential / V	% Mn^{4+}	% Mn^{3+}	% Mn^{2+}	Average Mn oxidation state	Adj. R-Square	Potential / V	% Mn^{4+}	% Mn^{3+}	% Mn^{2+}	Average Mn oxidation state	Adj. R-Square
1.43	89.7 ± 1.3	3.6 ± 3.2	6.2 ± 2.0	3.8 ± 0.3	0.9931	0.58	66.1 ± 1.3	29.3 ± 3.2	4.5 ± 1.9	3.6 ± 0.3	0.9931
1.38	87.6 ± 1.6	8.1 ± 3.8	3.7 ± 2.3	3.8 ± 0.3	0.9930	0.53	63.5 ± 1.3	31.4 ± 3.3	4.4 ± 2.0	3.6 ± 0.3	0.9931
1.33	88.8 ± 1.4	4.4 ± 3.3	5.7 ± 2.0	3.8 ± 0.3	0.9931	0.48	56.1 ± 1.3	41.3 ± 3.2	2.5 ± 2.0	3.5 ± 0.3	0.9931
1.28	91.9 ± 1.6	0.3 ± 3.8	7.4 ± 2.3	3.8 ± 0.3	0.9930	0.43	51.8 ± 1.1	46.7 ± 2.6	1.9 ± 1.6	3.5 ± 0.2	0.9931
1.23	91.1 ± 1.6	-1.0 ± 3.9	9.1 ± 2.4	3.8 ± 0.3	0.9930	0.38	51.5 ± 1.4	41.0 ± 3.3	7.7 ± 2.0	3.4 ± 0.3	0.9931
1.18	90.4 ± 1.6	0.7 ± 3.9	8.0 ± 2.4	3.8 ± 0.3	0.9930	0.33	32.4 ± 1.3	63.0 ± 3.3	4.3 ± 2.0	3.3 ± 0.3	0.9931
1.13	90.2 ± 1.3	0.1 ± 3.1	9.2 ± 1.9	3.8 ± 0.3	0.9931	0.28	33.1 ± 1.2	61.3 ± 3.0	5.7 ± 1.9	3.3 ± 0.3	0.9931
1.08	85.7 ± 1.3	6.8 ± 3.3	6.7 ± 2.0	3.8 ± 0.3	0.9931	0.23	22.1 ± 1.7	70.6 ± 4.2	7.0 ± 2.6	3.1 ± 0.4	0.9930
1.03	86.3 ± 1.5	6.9 ± 3.6	6.5 ± 2.2	3.8 ± 0.3	0.9930	0.43	23.5 ± 1.4	70.2 ± 3.4	6.6 ± 2.1	3.2 ± 0.3	0.9931
0.98	85.7 ± 1.5	7.5 ± 3.7	6.4 ± 2.2	3.8 ± 0.3	0.9930	0.63	40.0 ± 1.1	50.5 ± 2.6	9.1 ± 1.6	3.3 ± 0.2	0.9931
0.93	84.5 ± 1.5	8.5 ± 3.6	6.2 ± 2.2	3.8 ± 0.3	0.9931	0.83	64.7 ± 1.5	37.6 ± 3.6	-3.1 ± 2.2	3.7 ± 0.3	0.9930
0.88	85.4 ± 1.3	5.3 ± 3.3	9.4 ± 2.0	3.8 ± 0.3	0.9931	1.23	73.7 ± 1.4	24.8 ± 3.3	1.8 ± 2.0	3.7 ± 0.3	0.9931
0.83	79.0 ± 1.4	16.5 ± 3.4	3.8 ± 2.1	3.7 ± 0.3	0.9931	1.43	82.1 ± 1.3	13.1 ± 3.1	4.5 ± 1.9	3.8 ± 0.3	0.9931
0.78	82.2 ± 1.3	10.4 ± 3.2	7.2 ± 2.0	3.78 ± 0.3	0.9931						
0.73	77.6 ± 1.3	18.1 ± 3.2	3.8 ± 2.0	3.7 ± 0.3	0.9931						
0.68	74.6 ± 1.2	18.5 ± 2.9	6.2 ± 1.8	3.7 ± 0.2	0.9931						
0.63	70.3 ± 1.1	23.6 ± 2.7	5.2 ± 1.7	3.6 ± 0.2	0.9931						

Table S4. Results of the linear combination analysis of $LaMnO_3$ XES spectra by the references MnO_2, Mn_2O_3 and MnO at different potentials in a cathodic (blue) and anodic (yellow) scan.

Cathodic scan						Anodic scan					
Potential / V	% Mn^{4+}	% Mn^{3+}	% Mn^{2+}	Average Mn oxidation state	Adj. R-Square	Potential / V	% Mn^{4+}	% Mn^{3+}	% Mn^{2+}	Average Mn oxidation state	Adj. R-Square
1.43	77.4 ± 1.9	2.0 ± 4.6	18.6 ± 2.8	3.5 ± 0.4	0.9996	0.58	42.1 ± 1.1	49.1 ± 2.6	9.2 ± 1.6	3.3 ± 0.2	0.9999
1.38	82.0 ± 1.6	2.0 ± 3.8	14.7 ± 2.3	3.6 ± 0.3	0.9997	0.53	34.8 ± 1.1	56.4 ± 2.8	9.4 ± 1.7	3.3 ± 0.2	0.9999
1.33	78.0 ± 1.3	7.9 ± 3.3	12.2 ± 2.0	3.6 ± 0.3	0.9998	0.48	35.4 ± 1.2	46.7 ± 2.8	17.8 ± 1.7	3.2 ± 0.2	0.9999
1.28	74.7 ± 1.5	10.7 ± 3.7	13.5 ± 2.2	3.6 ± 0.3	0.9998	0.43	26.3 ± 1.1	54.6 ± 2.6	18.6 ± 1.6	3.1 ± 0.2	0.9999
1.23	71.4 ± 1.1	16.1 ± 2.8	12.0 ± 1.7	3.6 ± 0.2	0.9999	0.38	11.7 ± 1.1	72.0 ± 2.6	16.2 ± 1.6	3.0 ± 0.2	0.9999
1.18	66.1 ± 1.4	28.0 ± 3.4	5.8 ± 2.1	3.6 ± 0.3	0.9998	0.33	0.0 ± 0.0	83.5 ± 1.4	16.2 ± 1.3	2.8 ± 0.1	0.9998
1.13	68.7 ± 1.2	21.1 ± 3.0	9.6 ± 1.8	3.6 ± 0.2	0.9998	0.28	0.0 ± 0.0	76.1 ± 1.7	23.3 ± 1.6	2.8 ± 0.1	0.9997
1.08	69.4 ± 1.3	19.0 ± 3.2	10.7 ± 1.9	3.6 ± 0.3	0.9998	0.23	0.0 ± 0.0	74.5 ± 1.6	25.7 ± 1.6	2.8 ± 0.1	0.9997
1.03	67.4 ± 1.3	21.9 ± 3.3	10.1 ± 2.0	3.6 ± 0.3	0.9998	0.43	0.0 ± 0.0	68.8 ± 1.8	30.7 ± 1.8	2.7 ± 0.1	0.9997
0.98	67.0 ± 1.4	22.3 ± 3.4	10.5 ± 2.1	3.6 ± 0.3	0.9998	0.63	0.0 ± 0.0	81.7 ± 1.5	18.3 ± 1.5	2.8 ± 0.1	0.9998
0.93	63.3 ± 1.2	25.0 ± 3.0	11.6 ± 1.8	3.5 ± 0.2	0.9998	0.83	58.3 ± 1.3	29.1 ± 3.3	12.6 ± 2.0	3.5 ± 0.3	0.9998
0.88	63.3 ± 1.3	26.3 ± 3.1	9.9 ± 1.9	3.5 ± 0.3	0.9998	1.23	68.8 ± 1.5	23.2 ± 3.6	8.6 ± 2.2	3.6 ± 0.3	0.9998
0.83	59.8 ± 1.4	32.0 ± 3.3	8.1 ± 2.0	3.5 ± 0.3	0.9998	1.43	69.4 ± 1.4	26.0 ± 3.3	4.5 ± 2.0	3.6 ± 0.3	0.9998
0.78	48.2 ± 1.1	50.0 ± 2.8	1.8 ± 1.7	3.5 ± 0.2	0.9999						
0.73	59.3 ± 1.2	30.1 ± 2.9	10.7 ± 1.8	3.5 ± 0.2	0.9998						
0.68	52.0 ± 1.3	39.1 ± 3.1	8.9 ± 1.9	3.4 ± 0.3	0.9998						
0.63	45.3 ± 1.2	45.8 ± 3.0	8.8 ± 1.8	3.4 ± 0.2	0.9998						

Table S5. Relative energy shift and the best fit results from the structural analysis of $La_{1-x}Ca_xMnO_3$ at the Mn K-edge and at OCP. N is the coordination number, R is the interatomic distance Mn-O and σ^2 is the Debye-Waller factor. R_f is the R-factor, which represents the relative error of the fit and data. $CaMnO_3$: 2.7 < k < 12.2; 1.0 < R < 4.0. $La_{0.4}Ca_{0.6}MnO_3$: 2.8 < k < 11.1; 1.0 < R < 4.0; $La_{0.6}Ca_{0.4}MnO_3$: 2.8 < k < 11.0; 1.0 < R < 4.0; $LaMnO_3$: 2.6 < k < 11.1; 1.0 < R < 4.0

	Shell	N	R / Å	σ^2 x 10^3 / $Å^2$	ΔE_0 / eV	S_0^2	R_f
$CaMnO_3$	Mn-O	6	1.89 ± 0.01	0.8 ± 0.3			
	Mn-Ca	8	3.22 ± 0.03	15.2 ± 3.2	-1.4 ± 0.9	0.54 ± 0.04	0.027
	Mn-Mn	6	3.72 ± 0.03	4.5 ± 1.9			
$La_{0.4}Ca_{0.6}MnO_3$	Mn-O	6	1.89 ± 0.02	4.1 ± 2.0			
	Mn-La	3	3.47 ± 0.33	22.1 ± 11.8	-3.8 ± 1.6	0.60 ± 0.1	0.029
	Mn-Ca	5	3.17 ± 0.08	22.1 ± 11.8			
	Mn-Mn	6	3.65 ± 0.03	5.9 ± 2.8			
$La_{0.6}Ca_{0.4}MnO_3$	Mn-O	6	1.90 ± 0.02	4.0 ± 1.9			
	Mn-La	5	3.30 ± 0.04	8.5 ± 2.8	-2.9 ± 1.7	0.60 ± 0.08	0.029
	Mn-Ca	3	3.38 ± 0.08	8.5 ± 2.8			
	Mn-Mn	6	3.37 ± 0.16	22.6 ± 14.6			
$LaMnO_3$	Mn-O	6	1.93 ± 0.01	3.6 ± 1.2			
	Mn-La	8	3.35 ± 0.01	5.2 ± 0.8	-1.4 ± 0.6	0.57 ± 0.04	0.016
	Mn-Mn	6	4.02 ± 0.02	8.1 ± 2.3			

Table S6. Best fit results from the structural analysis of the first coordination shell of $La_{1-x}Ca_xMnO_3$ at the Mn K-edge and at different potential conditions. N is the coordination number, R is the interatomic distance Mn-O and σ^2 is the Debye-Waller factor. R_f is the R-factor, which represents the relative error of the fit and data.

Potential	Shell	N	R / Å	σ^2 x 10^3 / Å^2	ΔE_0 / eV	R_f
$CaMnO_3$ $S_0^2 = 0.54$; $2.6 < k < 12.1$; $1.0 < R < 2.0$						
1.40 V	Mn-O	5.6 ± 1.0	1.89 ± 0.02	1.9 ± 0.3	-1.2 ± 2.3	0.022
0.70 V	Mn-O	5.6 ± 1.1	1.89 ± 0.03	2.2 ± 0.2	-1.4 ± 2.4	0.027
0.50 V	Mn-O	5.6 ± 1.1	1.89 ± 0.03	2.2 ± 0.2	-1.4 ± 2.5	0.027
0.40 V	Mn-O	5.3 ± 1.1	1.90 ± 0.03	2.2 ± 0.2	-1.3 ± 2.5	0.027
0.20 V	Mn-O	3.2 ± 0.8	2.24 ± 0.05	5.2 ± 4.9	-1.5 ± 2.7	0.036
$La_{0.4}Ca_{0.6}MnO_3$; $S_0^2 = 0.60$; $2.8 < k < 11.2$; $1.05 < R < 2.0$						
1.40 V	Mn-O	5.2 ± 0.9	1.90 ± 0.02	2.1 ± 1.1	-2.4 ± 2.4	0.008
0.70 V	Mn-O	5.2 ± 0.9	1.90 ± 0.02	2.1 ± 1.4	-2.4 ± 2.3	0.008
0.50 V	Mn-O	4.9 ± 0.8	1.90 ± 0.02	2.0 ± 1.4	-2.6 ± 2.3	0.008
0.40 V	Mn-O	4.6 ± 0.9	1.90 ± 0.03	2.4 ± 0.9	-3.3 ± 2.8	0.010
0.20 V	Mn-O	2.4 ± 0.5	1.89 ± 0.03	2.5 ± 1.8	-3.5 ± 3.5	0.017

Table S6. Continued

Potential	Shell	N	R / Å	σ^2 x 10^3 / $Å^2$	ΔE_0 / eV	R_f
$La_{0.6}Ca_{0.4}MnO_3$; $S_0^2 = 0.59$; 2.8 < k < 11.0; 1.0 < R < 2.2						
1.40 V	Mn-O	5.4 ± 1.0	1.89 ± 0.03	3.3 ± 2.5	-4.6 ± 2.4	0.022
0.70 V	Mn-O	5.5 ± 1.0	1.89 ± 0.03	3.9 ± 2.7	-4.9 ± 2.6	0.023
0.50 V	Mn-O	4.9 ± 0.8	1.89 ± 0.03	2.9 ± 2.3	-4.6 ± 2.4	0.019
0.40 V	Mn-O	4.3 ± 0.8	1.88 ± 0.03	3.2 ± 2.5	-5.7 ± 2.6	0.023
0.20 V	Mn-O	3.6 ± 0.6	1.87 ± 0.03	3.7 ± 2.4	-7.7 ± 2.5	0.019
$LaMnO_3$; $S_0^2 = 0.57$; 2.6 < k < 11.1; 1.0 < R < 2.0						
1.40 V	Mn-O	6.1 ± 0.7	1.92 ± 0.02	3.8 ± 1.7	-3.3 ± 1.5	0.007
0.70 V	Mn-O	6.1 ± 0.7	1.92 ± 0.02	3.9 ± 1.6	-3.2 ± 1.3	0.006
0.50 V	Mn-O	5.9 ± 0.5	1.92 ± 0.01	3.5 ± 1.3	-3.5 ± 1.1	0.004
0.40 V	Mn-O	6.0 ± 0.7	1.92 ± 0.02	4.1 ± 1.8	-3.4 ± 1.5	0.007
0.20 V	Mn-O	5.9 ± 0.5	1.92 ± 0.01	4.0 ± 1.3	-3.8 ± 1.1	0.004
